# The complexities and contradictions of online eating disorder content: A qualitative evidence synthesis of young people’s views and experiences

**DOI:** 10.1371/journal.pdig.0001066

**Published:** 2026-10-09

**Authors:** Meena Khatwa, Rebecca Rees, Claire Stansfield, Vidhi Bassi, Ceri-Nicole Wilkins, Kelly Dickson

**Affiliations:** 1 EPPI Centre, UCL Social Research Institute, University College London, London, United Kingdom; 2 Young Advisors on Young People and Online Eating Disorder Content: A Qualitative Evidence Synthesis Project, Eppi Centre, UCL Social Research Institute, University College London, London, United Kingdom; University of Pittsburgh School of Medicine, UNITED STATES OF AMERICA

## Abstract

Eating disorders (ED) continue to be a concern worldwide. There is a growing focus on problematic discourse around ideal bodies as well as on how exposure to related content and spaces online might influence disordered eating and other ED symptoms. A qualitative evidence synthesis using thematic synthesis methods was conducted to explore young people’s lived experiences of online ED content. Studies were included if they provided, from 2007 onwards, views about online content from young people from OECD countries aged 11–25 years old who identified as having had an eating disorder, disordered eating or eating difficulties. This article focuses on findings from 13 included studies whereby three key themes were identified: Comparing – how online content was used to evaluate others and oneself; Curating – how young people manage this content; and Community – spaces for connection over shared experience. Participants across the studies described the complex ‘double-edged’ nature of online spaces, which can offer inclusion but can also be experienced as harmful. The synthesis outlined a lack of supportive offline mental health-promoting spaces as part of the context of young people’s online content use.

## Author summary

Eating disorders (ED) continue to be a major global issue, with focus on ideal body type, and food restrictions. This article explores ED symptoms and behaviour within the context of online spaces and how they might influence young people. The researchers conducted a qualitative evidence synthesis which uses thematic analysis to explore existing research data on this topic. The 13 studies included focused on young people aged between 11–25 from OECD countries. Three key themes, showing how young people interact online were, firstly ‘Comparing’ – judging their own bodies against others online; secondly, ‘Curating’ – actively controlling and filtering what they see; and finally, ‘Community’ – looking for spaces to connect over shared struggles. The main takeaway from this research was that online spaces are complex and can act as a double-edged sword by offering support but also causing harm. Young people often look for these online communities because they lack supportive mental health spaces in their offline lives.

## Introduction

The rise of online eating disorder content is a growing concern due to the increased use of social media platforms and online communities [1]. Though eating disorder (ED) symptoms can be detected from as young as six into late adulthood; peak onset predominately occurs in early adolescence as does increased use of social media and engagement in online settings [2–4]. There is a growing focus on problematic discourse around ideal bodies as well as on how exposure to related content and spaces online might influence disordered eating and other ED symptoms. While quantitative impacts have been explored through systematic reviews of existing research [5] there appears to have been no systematic attempts to review the qualitative literature on young people’s own experiences and understandings of this online content. Qualitative evidence synthesis is therefore needed to complement the existing quantitative evidence, centering young people’s own perspectives to capture the meanings they attach to this content and their motivations for seeking it. Qualitative evidence synthesis is defined as “a type of systematic review that brings together the findings from primary qualitative research in a systematic way” [6]. The main aim of a QES is to provide rich interpretations of already existing qualitative research and provide greater understanding across carefully selected studies.

This qualitative evidence synthesis adopted a broad scope driven by young people’s own definitions. Thus, online eating disorder content was defined as any material or exchanges identified by young people as holding the potential to influence their own or others’ views about body image or about behaviours related to body size, shape, or weight. Online media could take text, visual or audio forms and could involve social media, blogs or instant chat. Content could address size, shape or weight, or any behaviours related to these, including, but not restricted to dieting or weight loss. Pro-ED content is sometimes seen as actively encouraging disordered eating behaviours, with pro-recovery content aimed at support and promote getting better [7,8]. In this review, however, we were interested in young people’s own definitions of such distinctions [7,8].

We have considered eating disorders such as anorexia nervosa (AN), bulimia nervosa (BN), and binge eating disorder (BED), and how they present in different ways but are all characterised by distress or concerns about body image and eating [9]. Globally, it is estimated that over 3.3 million healthy lives are lost due to eating disorders, with increases in both the risk of suicide and premature death, with the highest mortality rates occurring in young people with anorexia [10–12]. Higher incidence of eating disorders is reported in girls and young women, however, emerging research with boys and young men has begun to identify specific disordered eating and weight gain behaviours related to body ideals that emphasise muscularity and leanness, and which can lead to elevated ED symptoms [13,14]. Living with an eating disorder can be highly disruptive, exerting a negative impact on a young person’s sense of self, relationship with others and overall quality of life [15,16]. Similar concerns have also been raised in relation to the amount of time children and young people spend in online spaces [17].

Complex interactions among bio-psychosocial and environmental mechanisms can also contribute to EDs in young people [18,19]. A key area of investigation explores the particular influence of sociocultural views about ‘ideal’ bodies [20,21]. Historically, traditional media (for example, television, films, magazines) have been theorised as a social arena that generate and reproduce discourses about ideal body images [22]. This has led to a large volume of empirical research on exploring the effects of mass media on eating disorders in children and young people [23,24]. In the advent of the internet, this literature has now extended to consider the impact of digital media on young people’s body dissatisfaction and disordered eating [25].

A distinctive feature of online digital media platforms (e.g., websites, online forums, and social networking sites), is their interactivity. They enable young people to be generators and receivers of personal and targeted content, often simultaneously [26], via digital media platforms (for example, Instagram, Tumblr, YouTube, Pinterest, Facebook and Tiktok) [8,27–29]. With the increase in new types of digital media, there have been growing concerns about the widespread existence of pro-ED communities [30] and their accessibility and expanding scope of ED content, which are no longer confined to websites and online forums.

The above rationale supported us to answer the following research question: What are young people’s views and experiences of online eating disorder content, and the influences it can have on their relationship with their bodies, weight control behaviours, and/or any other eating disorder symptoms?

## Methods

A review of research was undertaken which included a systematic map and two qualitative evidence syntheses (QES). The review protocol was published on the PROSPERO systematic review registry. The methods used for the QES, are summarised below, with additional detail provided in Table A in S1 Appendix and a technical report [31].

This paper only reports on the synthesis of qualitative studies of young people with lived experience of an eating disorder, disordered eating or eating difficulties. A second synthesis (not reported here) was conducted on studies where the sample consisted of young people who were not reported as having this lived experience.

### Eligibility criteria, search strategy and study selection

For studies to be included in the QES, they needed to use qualitative methods to investigate young people’s experience and views of online eating disorder or body-image content. The sample needed to be aged between 11–25 and identify as having had an eating disorder, disordered eating or eating difficulties. Studies also needed to be conducted in a member country of the Organisation for Economic Co-operation and Development (OECD), to help findings be applicable to a UK policy context and published in English from 2007 onwards. A comprehensive search was undertaken to identify research on body dissatisfaction, disordered eating or over-exercising symptoms and behaviours as well as online content and young people. This involved searches of 15 scholarly bibliographic databases in health, psychology and social science, eight online resources and search engines, four online journals, and forward and backward citation searching (see Box A in S1 Appendix). Two update searches were run (the last in October 2024) to seek additional, and more recent studies.

Search results were imported into the review software, EPPI-Reviewer 4 [32], which was also used to manage all subsequent stages of the review described below. References were de-duplicated, then the research team screened initially on titles and abstracts and accepted studies for review based on the eligibility criteria. We piloted the exclusion criteria by comparing decisions in groups of two or more reviewers using worksheets with guidance notes on a sample of 20 records. A further sample of records were screened by reviewers independently and differences resolved by discussion or consulting with a third reviewer. When agreement was adequate (90–95%) for this second sample, the remaining citations were screened by a single reviewer. Full reports were obtained for those references and judged as meeting the inclusion criteria or where there was insufficient information from the title and abstract to assess relevance. The reports at this stage were all screened by two reviewers, who again met to discuss any differences.

### Characterising the studies and extracting findings

The coding tool used for characterising studies was devised from those used in two previous reviews of related topics (online activity and adverse experience among young people) [17,33]. Two reviewers [MK & RR] piloted and further developed the tool for use in this study. Each study was then coded independently by the two reviewers who then reached a consensus to produce a final agreed coding. The findings of each study were identified from within the results sections of included reports, again independently by these two reviewers. Findings that were labelled by authors as relating to participants who did not meet our inclusion criteria, either because of age or lack of lived experience, were not extracted for the synthesis.

### Quality assessment of primary studies

The appraisal of the primary studies was done independently by two of the reviewers [MK, RR], who then reached a consensus. The allocated studies were critically appraised against six sets of questions used in previous QES reviews (see Box B in S1 Appendix). These questions explored each study’s approach to: (1) sampling; (2) data collection; and (3) data analysis; (4) the extent to which the study was grounded in the data presented; (5) the breadth and depth of the findings; (6) the extent to which the study privileged young people’s perspectives. Study quality was summed up in terms of reliability (which considered the judgements made using criteria 1–4; and usefulness (which used questions 5 and 6).

### Data synthesis

Data was synthesised thematically [34] (see Table A and Table C in S1 Appendix), and included qualitative data consisting of participants’ quotes and authors’ interpretation taken direct from the findings sections of the primary studies. The process was inductive and involved line-by-line coding by the two reviewers [MK & RR] and was carried out independently using the coding function in EPPI-Reviewer. By using an iterative process, the coding of the data was then organised into themes whereby higher-order themes were identified through repeated individual coding and follow-up discussions between these two reviewers. The discussions between the reviewers led to the synthesis being divided into two parts due to the complexity and breadth of the data. The team first worked on coding and drafting a narrative synthesis for 13 studies of views from young people with lived experience of EDs. Two of the young people who had participated in the workshop [VB & CNW] also worked on this synthesis to help incorporate the findings of the four most recently published studies.

## Results

After the removal of duplicates, a total of 10484 references were screened on title and abstract and 693 references were rescreened on a basis of the full-text reports. Fig 1: PRISMA diagram illustrates the review’s screening process. A total of 13 studies, reported in 19 papers were included in the review. For additional details see PRISMA Checklist in S1 Appendix.

**Fig 1:**
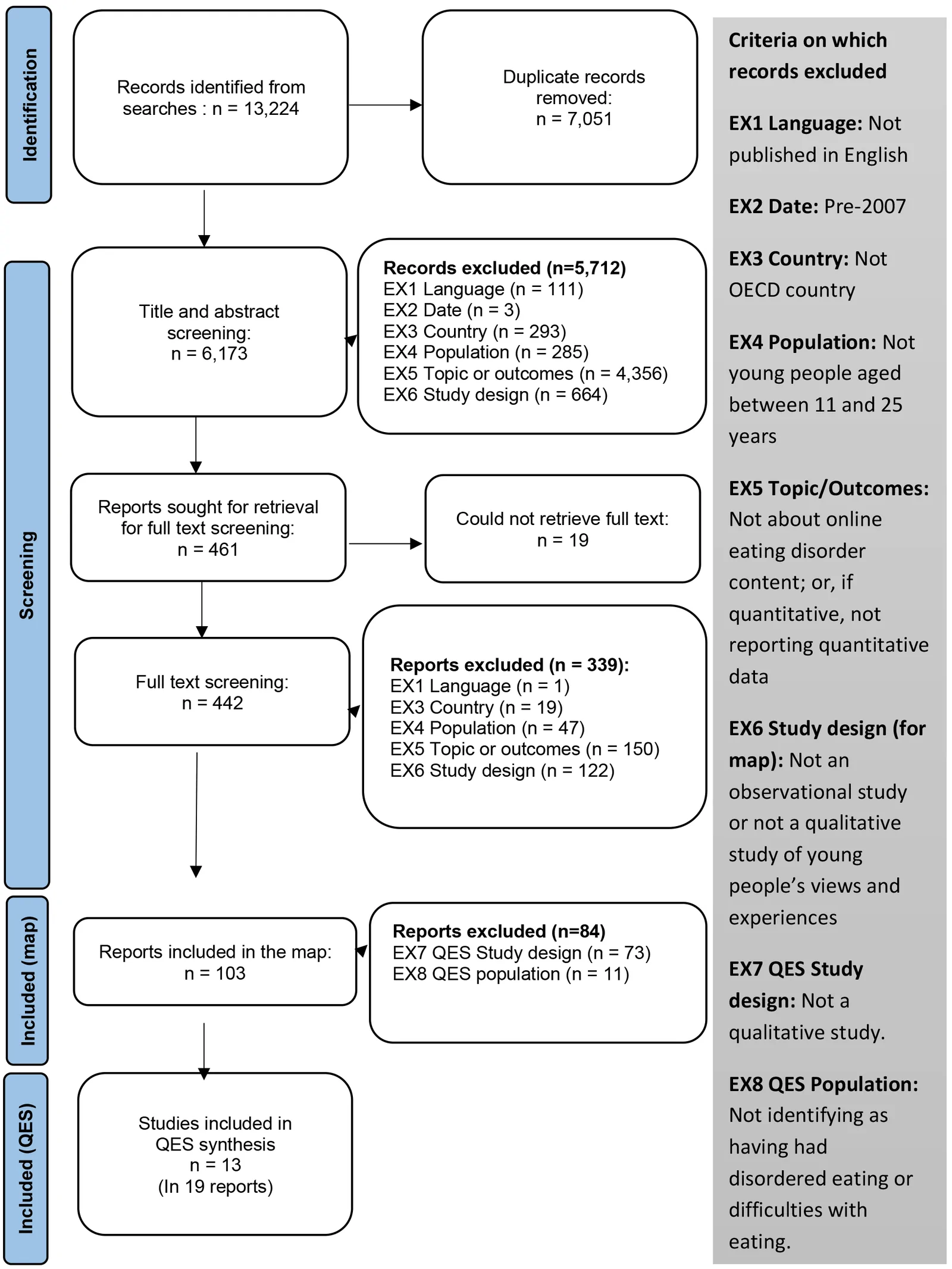
PRISMA diagram.

### Focus of included studies

The 13 studies had all been published since 2017, indicating this is a relatively new field of qualitative enquiry. A range of OECD countries were focused upon, including the USA (4 studies), the UK (2 studies), and Belgium, the Czech Republic, Italy, New Zealand and Sweden (1 study each). Two additional studies recruited participants from across several countries.

In terms of the kinds of young people participating, only one study reported clearly that it involved young men with lived experience of EDs, and only one participant in this study fell within this review’s age range focus [35]. A range of ages were represented, with seven studies’ samples including participants aged less than 18 [36–42]. Although studies collected information about class and ethnicity, exploration of disadvantage, such as how ethnicity or socioeconomic status might intersect with subjective experiences of engaging with online content, was not a focus in any of the studies. In terms of content, four of the 13 studies explored pro-ED focused content, or content perceived as harmful, one focused upon recovery-focused content, while the remainder focused on more general content promoting fitness, health and/or body-image ideals, or were open to discussion of any of these content types. In terms of online platform types, one study focused upon TikTok and four on Instagram (one also exploring Snapchat), while others focused upon blogs, the use of selfies and social media more generally. Table 1 below provides a snapshot of study characteristics. For further details on the selected studies please refer to Table A and Table B in S2 Appendix

**Table 1:** The population and topic focus of studies included in the review.

Author	Study population(location/sample size and ED-related characteristics)*	Study topic(content and platform type)
**Dashow, 2021**	• USA; n = 12 • Had ED recovery focused accounts	• Content focus: Recovery • Platform: Instagram
**Eikey & Booth, 2017**	• USA; n = 16 • Formally diagnosed with ED and/or have had symptoms of anorexia or bulimia. Most reported being in recovery	• Content focus: Body Image Ideals, Fit, Thin • Platform: Websites, Instagram
**Faccio, 2024**	• Italy; n = 30 • With a diagnosis of an ED, and in the early stages of hospital treatment	• Content focus: Body Image Ideals, Self-representation • Platform: Instagram and Snapchat
**Firkins, 2017**	• UK & USA; n = 3 • A history of eating difficulties and previous regular use of pro-ED sites now reduced or stopped altogether	• Content focus: Pro-eating disorder • Platform: Websites, social media
**Hockin-Boyers et al., 2021**	• UK; n = 19 • A history of ED, now weightlifting during recovery	• Content focus: Body Image Ideals, Fit (transformation photos) • Platform: Instagram
**IPSOS and Tonic, 2024**	• UK; n = 8** • Self-identifying with an ED, with at least six months since discharge from an ED support service and without an active ED diagnosis	• Content focus: Content perceived as harmful by participants • Platform: Not pre-specified
**Mincey, 2019**	• USA; n = 10 • Accessing and using pro-eating disorder online media at the time of the study. All recounted previous experience with an ED or disordered eating.	• Content focus: Pro-eating disorder • Platform: Websites, social media (forums)
**Nikolova & LaMarre, 2023**	• New Zealand; n = 8 • In recovery/on a journey to recovery from disordered eating and using Instagram 3+ times a week	• Content focus: Health and fitness • Platform: Instagram
**Praet, 2024**	• Belgium; n = 7 • With a DSM-5 diagnosis of Anorexia, restrictive type, recruited through a paediatrics department	• Content focus: Body Image Ideals, Pro-eating disorder, Recovery • Platform: Social media
**Saunders et al., 2020**	• USA; n = 15 • Self-identifying as in ED recovery	• Content focus: Body Image Ideals: Beauty/Attractiveness (selfies) • Platform: Social media
**Sjöström, 2024**	• Sweden; n = 14 • With a DSM-5 diagnosis of Anorexia, recruited through an ED clinic.	• Content focus: Recovery • Platform: TikTok
**Smahelova et al., 2020**	• Czech Republic; n = 30 • Experience (current or past) of an ED	• Content focus: No restriction • Platform: Social media, Websites
**Yeshua-Katz and Martins, 2013**	• USA, UK, Australia, New Zealand, the European Union; n = 33 • Running a Pro-anorexia blog, small number reported being in recovery, most reported living with an ED	• Content focus: Pro-anorexia • Platform: Social media (blogs)

* With the exception of one young man in Mincey (2019) and an unspecified number in IPSOS and Tonic (2024), all participants were female. ** Some of the findings in this study could not be used for our review as participants included young people who did not necessarily have previous lived experience of an eating disorder, disordered eating or eating difficulties. Some were described as having experienced self-harm, suicidal ideation, anxiety and/or depression, or as having encountered content that they perceived as harmful for these conditions.

### Quality of studies and consultation with young people

The quality of studies is summarised in Table 2. All but one study was judged to be of at least medium reliability and/or usefulness for the purposes of this qualitative evidence synthesis.

**Table 2:** Quality assessment of studies: reliability and usefulness.

Author	Reliability			Usefulness		
	High	Medium	Low	High	Medium	Low
Dashow 2021		✓			✓	
Eikey & Booth 2017			✓			✓
Faccio et al., 2024		✓			✓	
Firkins et al., 2017		✓		✓		
Hockin-Boyers et al., 2021	✓			✓		
IPSOS 2024		✓				✓
Mincey 2019		✓			✓	
Nikolova & LaMarre, 2023		✓			✓	
Praet et al., 2024		✓			✓	
Saunders et al., 2020		✓			✓	
Sjöström et al., 2024	✓			✓		
Smahelova et., Al 2020	✓			✓		
Yeshua-Katz & Martins 2013		✓		✓		

In a first consultative workshop with young people before searches were finalised, there was a discussion of the kinds of online eating disorder content and language that young people might be using and what mattered most for young people about this content. The young people discussed emerging findings from the synthesis at the second workshop. They identified aspects that they considered important but that might have been missed by the research team when coding the included studies. These included the use of this content for self-validation, and sites that present as focused on ED recovery. The reviewers added detail about both to the synthesis, aided by the addition of new studies found after update searches were run.

### Thematic synthesis findings

After carrying out an extensive iterative coding exercise by the two reviewers (MK & RR) of each study we began to identify common overarching themes from the synthesis where young people expressed similar experiences or behaviours. We began initially with five overarching themes which included: identities and journeys and another theme on triggers. As there was not sufficient data to justify these as standalone themes, we decided to incorporate them into the following three distinct overarching themes: (1) Comparing, which illustrated how young people use content to evaluate their own and others’ bodies and behaviours. (2) Curating, which explored how young people manage and create content, and lastly (3) Community, which examined how young people were drawn to and interacted in online social spaces because of opportunities for connection with others with similar identities.

### Comparing

All 13 studies explored how young people used ED content found on diverse online platforms to evaluate others and themselves [35,37–48]. This theme and the sub themes that follow cover a range of complex behaviours and experiences which include types of content used to compare such as weight loss and body image; how content which is easily accessible can trigger negative thoughts such as failure but also feelings of affirmation. It is also pertinent to highlight that young people’s experiences were influenced by where they were in their eating disorder journey, and accounts of online comparisons in recovery were sometimes positive, but not always.

### *Comparisons are constant – “seeing thin people on my feed helps”* [44]

There were many types of content posted online which illustrated a comparison context. This included: calorie counts or the size of food portions, and accounts of exercise taken [42,44], popularity which resulted in the form of views or likes [48], as well as images of bodies [37,42,44,45,48]. The young woman quoted in the above heading was described as in recovery and expressed how she had previously felt reassured by looking at others’ online content, e.g., Thinspiration on Instagram, as well as others contributing to her feed. This content could be found on platforms which included anorexia-focused and pro-recovery forums [35,37,44,45] but also on social networking platforms including Instagram [44], Snapchat (e.g., [39] and TikTok [42] as well as social networking sites not specified by study authors [41,42]. The 13 studies also revealed that ease of access to undesirable online content could lead to feelings of over exposure. Young people used terms like “constantly” [36,44], “bombarding” and “attacking” [49] and “[getting] hooked” [42] when describing this experience. One study’s authors [47] described how users in recovery were critical of algorithms within Instagram which could ‘misinterpret’ what young people wanted to see, treating diet content and body-positivity as synonymous (e.g., sending diet-recommendations as opposed to encouragement not to moralise food). These experiences coupled with the ubiquitous nature of comparative online words and images meant they were also implicated as potential triggers of negative emotions [35–39,41,42,47]. One group of authors noted that comparisons of many sorts (food consumption and thinness, but also social success) were particularly troubling for some young people in their recovery or treatment phase of the ED journey, whereby comparing to others led to weight loss behaviours and “pulled them back to the illness” [37,49]. As one young woman put it, “I watched a lot of food videos… There were many food videos and calorie counts, even though these were people trying to show that they could eat, recover, and that life was worth living. I just focused on the food and compared myself” [42]. Relatedly, young people described as pro-ED bloggers [38] reported feeling concerned about potentially triggering “an ED in other young girls or people trying to recover from an ED” [38].

### *Comparison can affirm but also undermine sense of self – “this person is skinnier than me so she’s sicker than me”* [35]

The viewing of online content could conjure up complex feelings of both failure and success. The young person quoted in this section’s heading, was one of several in one study who recounted online comparisons that had left them feeling insufficiently sick to deserve support [35]. Online comparisons could influence and lead to feelings of low self-esteem whereby, when viewing others’ accounts of their behaviours or bodies, participants felt triggered [40,41] or like “losers”, in that “they are doing a better ‘job’ of having an eating disorder” [35]. The potential for “negative inspiration” was also emphasised, where “pro-ana blogs represented EDs as a desirable lifestyle” [49]. However, the emotional impact of looking at online content when comparing could also be positive, when in recovery. Young women reported seeing online body transformation, where others went from underweight to deliberately gaining muscular weight, or “becoming body builders”, and accounts documenting self-transformation were celebrated [46]. It was striking that the feelings expressed about comparisons by young people very much depended upon where they were in their eating disorder journey, and being generally more positive for those identifying as in recovery [35,36,48,49].

### Curating

This overarching theme captures different levels of engagement which go beyond merely observing and include the management and creation of content. The theme considers activities such as: use of hashtags or other means for seeking out certain content producers or types of content; liking and providing feedback to others’ individual posts; and otherwise interacting with existing content. Young people in the studies also deliberately created new material, such as uploading images on Instagram (annotated or otherwise), running Instagram recovery accounts, posting questions and responses in specialised forums, and writing blog articles. The following sub-themes highlight the key findings about, in turn, what is interpreted as curation, what young people engage with and why, and how this impacts behaviour and lived experiences of ED.

### *Seeking out and creating content that is authentic – “I think it gives us more access to real people”* [36]

One element of curation that resonated with young people was the seeking out of online ED content that they could relate to alongside their own journeys. Young people searching for pro-ED material expressed a desire for following “concrete – real people with ED and their stories” [37]. In part this aided participants’ motivation for recovery because they had realistic examples “where real people [on social media...] are having the same thoughts” [36] rather than in the past where most content that was found in magazines was of celebrities only. The value of authenticity is also shared by blog creators who in one study, were keen to: “share and declassify,” or to open up about themselves [49]. This sharing included young people reporting on their whole life and not just their ED behaviour; they positioned their posts “within their everyday life and family context” [37]. The complexity of this act is indicated by one young woman, who shared: “I often refer to how my blog is my true self and how in real life I have to act and hide my true feelings[...]. My blog is who I am backstage when I’m stripped of the make-up and costume” [38].

Positive experiences of authentic content were however countered by views of negative impacts associated with inauthentic content, such as image manipulation [41]. The unhelpfully selective sharing of content was implicated in concerns about “people lying to themselves  ... and lying to others that they were recovering” [43,49].

### *Managing and interacting with others’ content – “it’s not a good or a bad thing, it’s a tool”* [36]

Young people described diverse ways of interacting with content. The young person in the quote above continued, “you can either… put out negativity, …or you can use it for good” [36]. A response to an individual’s post could be seen as a signal of support by young people, whether this was in pro-ED discussion sites [35,37] or in recovery-focused discussions [44,46,48]: “If I have a friend who lost a lot of weight. I’ll always comment … “you’re on a good path, you’ve done this in a healthy way” [44]. However, some responses aimed to encourage self-reflection. For example, in response to transformation photos on Instagram, one participant in recovery recalled posting how weight loss didn’t make a person happier [46].

Some young people were selective with who they followed and unfollowed [36,42] when content was considered unhelpful or, as one young woman put it, when “what you’re posting isn’t contributing positively towards my mental health” [47]. However, young people also commented upon the intensity of interactions and the amount of time spent in exchanges, which could both be high [37]. All participants in one study reported unintentionally encountering algorithmically recommended harmful content [40]. They also reported being wary when seeing extremely low-calorie diets or fasting but found it difficult to distinguish more generally between healthy lifestyle and pro-ED content. Others were careful with the platform’s ‘like’ function [42]. However, young people also identified reduced control over triggering content, reporting feelings of addiction [41], as well as algorithmic content coming “from nowhere”, and requirements for “endless scrolling” [42]. Others found it difficult to “filter out” things they didn’t want to see or disconnect from friends who demonstrated “toxic behaviour” [47].

### Varied motivations for generating new content – “to seek others that I could tell my story to” [44]

The studies revealed there were many motivations/factors for creating new content across different platforms (content other than annotations or direct responses to others’ material). For instance, bloggers who were living with ED placed an emphasis on producing content as a means of self-expression, gaining support (not feeling alone), coping with stigma and raising awareness of shared lived experiences [37,38,44]. Online spaces allowed young people to share their own ED stories with less danger of being judged: as one young woman put it “having an ED was so socially unacceptable that it pushed me to seek others that I could tell my story to” [38]. Describing the therapeutic properties of disclosure, one young woman noted, “there really happened an extreme disclosure and I think it was one of the most important steps (for recovery) … by putting it down in written form, and actually identifying with it  ... It’s like to say it out loud” [37].

Writing and sharing recovery content not only helped the bloggers but was seen as helping others. As well as self-expression through written content, young people also described the sharing of photos of visually appealing healthy meals or body transformations [46]. For example, young people who were weightlifting as part of their recovery reported comparisons between their own before and after selfie photos to be helpful, one commenting, “when I look back at what I used to look like and what I look like now it helps me be, like, ok. I am going in the right direction and other people can see it” [46]. Some pro-ED bloggers also reported writing to counteract inaccurate portrayals of EDs, to “make them realize that it’s not what everybody makes it out to be” [38].

### Community

The findings in this theme provide insight into why young people are drawn to online virtual spaces, whereby a sense of community connections is evoked through shared identification, experiences, aspirations, and where specialised information can be identified. However, these ED communities also appear to be complex spaces, which can be both helpful and harmful, thus constituting a double-edged sword. Again, it was evident that a young person’s level of engagement was influenced by their ED journey. For instance, in the early stages an individual might be curious or are seeking connections. Or perhaps further along the way there could be impacts on physical and emotional wellbeing, and disengaging could be complicated.

### Combatting loneliness and being misunderstood – “nobody normal understands why you want to starve yourself” [38]

The attraction of online communities was referred to in 11 of the 13 studies, where young people with experience of EDs revealed feelings of isolation, loneliness and being misunderstood by the offline world [35–38,41–45,47]. In contrast, these young people described how the online world offered spaces that were less judgmental with the potential for both instructive and emotional support [50], and meaningful connection “with other people, like-minded people, so a sense of community… Others who have had a similar experience to me, and whose viewpoint or worldview aligns with mine” [47].

It seemed that young people felt there was no alternative for them when in a disordered state, and that the offline world did not offer what they needed [35,37,38,42,45]. As one young person put it, “I almost felt that I was an alien that I just didn’t fit in anywhere… I think it’s where I belonged and that’s where I felt comfortable... I didn’t feel like I was in turmoil there” [45]. Another appeal of online communities was anonymity, this was described as a key driver of engagement where there were “no visual attachments” and was therefore “easier to converse without feeling threatened” [38]. However, anonymity was also mentioned as a way that people offering help could “degrade” others [40] and was thought to help “radicalize some people’s opinions” [49].

Some studies also highlighted limitations to connections within ED-related communities. For example, one young woman expressed that it was “not a healthy acceptance” when she had been in a disordered state. One young person, reflecting upon being part of a fitness recovery community, reported, “I don’t want to be, you know, the ‘eating disorder girl’ ” [46]. Elsewhere, young people in recovery who reflected back described that the pro-ED platforms “could keep them in isolation”. In this study, all but one of the participants who had used pro-ED sites reported no longer using them [37].

### Sourcing strategies – “I was only interested in how to endure so I did not have to eat” [37]

This quote and others across the studies illustrate how young people turned to online communities for information and learning. Pro-eating disorder sites were described by participants as spaces where strategies were shared by more experienced users, such as how to conceal ED symptoms, and suppress appetites, and otherwise bring about weight loss [35,37,38,41,45]. There was also reference to advice about avoiding “suffering from the condition” [35,37,38,45]. In one study, participants characterised this form of advice as harm reduction, and provided illustrations, for example: “...not brushing your teeth because you would brush the stomach acid around your mouth, and you’d rot your teeth faster. Things like that [are]...probably why I still have all of my teeth” [35].

Young people described other kinds of learning from online ED content, [35,37,38,45,47]. Specifically about the development of eating disorders [35], how to talk with others about EDs [47], self-acceptance and taking a healthy approach [42], as well as the recovery process [44]. Recovery testimonials were seen as having potential to support transformation [41,42].

Despite use of the term community, discussions of support and learning in all but two cases made no reference to anything other than individual actions. Young people with Instagram recovery accounts said they helped hold them accountable but said no more [43]. Elsewhere, one participant referred to mentoring others and having been awarded by her peers the status of “advanced warrior... in recognition of her Pro-Ana successes” [45]. In another study there was reference to the role of a moderator who maintains pro ED webpages, and how so-called Ana Coaches “would be called out in the thread”, presumably as a means of alerting others to danger [35].

### Online communities are a double-edged sword – “a thin line between receiving support and being triggered” [42]

This final sub-theme describes different initiations, and degrees of interaction within the online communities and how these intersect with the young person’s ED journey, emotions and behaviour. To add to the positive potential of avoiding isolation and learning for recovery described above, young people identify how online groupings can have negative impacts and how, at times, their online and offline journeys are nonlinear.

In terms of journeys, young people described different experiences of seeking out pro-ED content and making connections [40]. This could begin with “lurking” or being “curious” [35]. Others described primarily “looking for healthier recipes” [45], or how pro-ED content “popped up when … looking for other weight-loss things” [35]. Young people also talked about the influence of ED content on the pathway towards a more severe eating disorder. People posting content were also initially found and then followed through keyword searches [40]. Following a YouTube channel enabled one young person’s eating disorder to “bloom” [45]. One author described participants as having gone “deeper into illness … thanks to the anonymity, the presentation of extreme cases, and the safe environment where visitors are supportive and nice to each other” [37]. Later on in their ED journeys, half of those with Instagram recovery accounts in one study felt they had been problematic for their own recovery and several noted that the pro-ana community was present even within the recovery community [43]. In another study one young person described engaging with personal recovery accounts as "very unhelpful", and her having to “stay clear of them since realizing at the very start  ... this is not good on any level” [47]. One young woman described how her use would shift back and forward in a cycle between intense and frequent visits for ideas and support and stepping away to avoid “listen[ing] to everyone else’s problems” [45]. This was particularly pertinent for people in recovery who still reported using pro-ana sites in various ways, such as revisiting to look for friendship [45].

## Discussion

### Summary of findings and cross cutting themes

The main objectives of this study were to collect, analyse and present evidence on young people’s experiences and views of engagement with eating disorder-related material on online platforms. As far as we are aware, no study has previously explored studies of young people’s views on what kind of influences online eating disorder content has on their relationships with their body, weight control behaviours, and/or any other eating disorder symptoms.

This synthesis of 13 studies of participants with direct experience of an eating disorder, disordered eating or eating difficulties identifies diverse practices of comparing and curating online, and an emphasis on the value and challenges of being part of online ED-related communities. It starts to unpack the potential impacts online ED content can have on these young people. Recent quantitative primary studies have shown that social media platforms can be spaces of self-compassion and empowerment [51] but have also seen participants reporting the disadvantages and advantages of social networking [52].

Reduced levels of interaction with the offline world and a lack of external support/provision seemed to be a central experience of these young people. Online communities hold the potential to offer refuge from the stigma and judgement of the offline world and to fulfil a need to belong and be understood. A 2015 study using a netnographic approach to explore posts within youth moderated online ED forums which also echoed this notion in its identification of a “trusted environment for individuals to talk about isolation, fear… mutual support and acceptance” [53].

The use of pro-ana blogs was seen to provide emotional support, as other studies of online posts have shown [54,55]. Young people described embracing spaces where they could learn, comfortably self-evaluate, and share insights with others as to their shifting ED identities and therefore strengthen bonds [27]. However, this is also juxtaposed with young people’s awareness of associated dangers. Pro-ED communities had been experienced as helpful but also damaging by young people.

The potential for online content to trigger unwanted behaviours seemed particularly pertinent in this group. Other studies have also found similar findings on triggering content [56]. Our synthesis of studies of young people’s own experiences complements theory that highlights how being subjected to an external objectifying gaze can make women more likely to engage in self-objectification, increasing risk for body image disturbance and ED pathology. [57]

A level of awareness of potential triggers when producing and uploading content is also referred to by bloggers in one of the reviewed studies. This suggests a degree of responsibility and a perspective among online content producers that would benefit from further investigation. This review’s findings, however, should already illustrate for practitioners and policymakers how young people are active agents and producers of such content and not just passive consumers.

Our findings suggest that policy interventions could focus upon the routine exploration by professionals of the role of digital online platforms as a tool that can support or hinder recovery from eating disorders. This is already echoed in NICE guidelines for the treatment of eating disorders, in the assessment of the wider social community, including the internet and social media, alongside the impact of the home, education and work, throughout treatment [58]. We argue that this assessment could be extended to consider and explore the impact of online communities for instance, examining the formation of ED identities and degrees of engagement. Similarly, digital literacy interventions, particularly when related to online harms, could support young people to understand not only that engagement with certain types of content can be detrimental to their mental health, but also that identifying the line between helpful and unhelpful is subjective and could require outside help to decipher (for example, by consulting with family, friends or teachers). Given that young people often go online to seek information and support, there is not only scope for online platforms, such as social media and networking sites, to direct young people towards professional help, but also for platforms, including mobile applications, to become tools for increasing the accessibility of help for young people.

Furthermore, the awareness demonstrated by young people about online content and inauthenticity, competitiveness and self-critiquing requires further scope for alternative-evidence based prevention/intervention approaches (for example, self-compassion) [59].

The findings from the QES also illustrate the contradictory and very complex nature of online experiences, thus evoking the idea of these environments comprising a ‘double-edged sword’ [60,61]. This phrase is discussed elsewhere [62], in relation to “helping or harming one’s mental health and tendency towards eating disorders”. The shifting impact of eating disorder content across different stages of the disorder adds yet another layer of complexity. An understanding of this complexity could inform the work of social media providers in developing more effective detection and reporting tools for online eating disorder content on their platforms, as well as educational materials for users. The studies also emphasised the importance of authenticity and how material might present distortions of reality. The sense of endless opportunities for body image-related comparisons and negative consequences suggests considerable commonality of experience for young people as a whole. All of these themes could benefit from further study with young people.

The salience of community in young people’s engagement with online content could be incorporated into existing conceptual frameworks to enhance understanding of fluid and fluctuating engagement with content [63,64]. The observation of content, and the curating acts of following, commenting, and contributing content, are all likely to have different meanings for young people when done in the context of entering, engaging within, and leaving virtual social spaces where shared identity seems to be so key.

### Limitations of this review, current research and future research

This review has revealed multiple terms used in the literature to describe the many complexities of eating disorders and body image. As reviewers, we have attempted to represent views and experiences by adhering to the ways that these have been expressed and reported in the included studies. For example, terms such as ‘Recovery’ were identified as a recurring theme as a main topic focus or what was shared by the young people. However, there was very little discussion by the young people studied of the use of recovery sites, or in-depth discussion of how different content and sites could impact on recovery.

The synthesis highlighted the gender imbalance in the studies, with only one study out of 13 providing a young man’s perspective. This suggests that there are evidential gaps here and that further investigation into male experiences of EDs and body norms is needed. There was also no demographic data reported on sexuality or exploration of the experiences of the LGBTQIA+ community, despite body image being a significant issue for this community. Although there were five studies with participants from different ethnic groups, the number of participants taking part was small. It was also difficult to discern from the data the ethnicity of individuals and whether that this had any bearing on their lived experience. This lack of disclosure could be to protect the individual. However, it would be useful for future research to collect data from ethnic minority participants and members of the LGBTQIA+ community purposively, to explore whether their cultural/religious upbringings or identities play any role in the types of ED content they engage with and the nature of this engagement.

As we see an ever-increasing number of younger children using digital devices and gaining access to online platforms, it seems increasingly important to investigate this within the ED context. The more recent literature made steps in including young people aged 17 and under. This is an extremely sensitive topic and considerable attention to ethical care is needed when exploring lived experience of eating disorders, especially among younger young people.

Finally, in terms of media platforms, the studies in the review primarily analysed data about Instagram, with only one study each exploring TikTok and Snapchat. However new platforms are emerging all the time and there is a need for both researchers and reviewers to somehow keep abreast of the platforms young people are actually using.

All of the above could be considered limitations to the review, however, all reviews are necessarily limited by the studies that are available. It is worth noting that study authors in this field face ethical challenges when seeking to carry out research with young people who are in the midst of an eating disorder.

One additional limitation relates to time; more recent primary studies may now have been published, as this is a rapidly growing area of research [31]. Digital platforms, distinct from those discussed in the research identified to date, may now be preferred by users more. Young people’s perspectives may also be influenced by rapidly changing policy environments [65], which include legislation to ban social media access in some countries [66,67].

### Conclusion

This is the first qualitative evidence synthesis to examine online eating disorder content and provides an important account of how young people experience online eating disorder and idealised body image content. The findings highlight synergies in engagement around the key themes such as ‘community’ which had not yet been explored to any depth within the context of online eating disorder content. This paper also draws attention to the need for further engagement with this complexity, given the ongoing relationship young people have with digital online platforms and the subjectivity of their experiences in those spaces. New and existing policy interventions focused on supporting young people in this area could consider whether professionals routinely explore the role of online platforms as a tool that can support or hinder recovery from eating disorders. There is much potential for initiatives where those with lived experience of eating disorders and the use of online content work with health professionals and researchers to co-design online spaces and campaigns [68]. Digital literacy interventions, particularly when related to online harms, could support young people to understand not only that engagement with certain types of content can be detrimental to their mental health, including body image and self-esteem, but also that identifying the line between helpful and unhelpful is subjective. Therefore, it can be difficult to determine and could require outside help to decipher (for example, by consulting with family, friends, or teachers). Given that young people often go online to seek information and support, there is not only scope for online platforms, such as social media and networking sites, to direct young people towards professional help, but also for platforms, including mobile applications, to become tools for increasing the accessibility of help for young people. The design of digital interventions could also be informed by how young people use online spaces, to develop a person-centred and strength-based approach to addressing eating disorders and body image concern. Furthermore, the awareness demonstrated by young people about online content and inauthenticity, competitiveness and self-critiquing requires further scope for alternative-evidence based prevention/intervention approaches (for example, self-compassion) [69].

## Supporting information

S1 Appendix Full methods and search strategy. (ZIP)

S2 Appendix Details of included studies in synthesis. (ZIP)
