# Supplementary material for: The complexities and contradictions of online eating disorder content: A qualitative evidence synthesis of young people’s views and experiences
Source: PLOS Digit Health. 2026 Oct 9;5(10):e0001066. doi: 10.1371/journal.pdig.0001066 (PMC13652209; doi:10.1371/journal.pdig.0001066)
Supplement: Supplementary file 1 [file pdig.0001066.s001.zip › S1_Appendix/TableA.docx]

**S1 Table A**

Overview of methods for the review

| **Review consultations and collaboration / Review stages / Information management** | **Overview** (cited references are presented in full at the foot of this table) |
| --- | --- |
| **Review consultations and collaboration** | |
| **Consultation work with review commissioners and policy and practice stakeholders** | Review facility team members (KD, RR, MK and JT) met with the Department of Health and Social Care mental health policy team throughout the research process to ensure the review remained closely aligned with their needs and emerging policy requirements. We discussed with this team the scope of the protocol, the findings from the systematic map and the interim findings of the qualitative evidence synthesis.  A multidisciplinary advisory panel was set up to inform and guide the review. It consisted of professionals specialising in child and adult eating disorders. The group met once, at the stage of emerging findings about the nature and quantity of research evidence identified by the map to support the scope and shape of review. |
| **Consultation workshops with young people and collaboration work with two young advisors** | We held two online workshops to consult with a group of eight young people with lived experience of online eating disorder content to help ensure the review explored areas of relevance to young people. This work was supported by a specialist in the active involvement of young people in research. Workshop 1 was held when coding work had started to identify the extent and nature of existing research studies. Workshop 2 was held when findings were starting to emerge from the qualitative synthesis. Workshop discussions were informal and open-ended but were structured around the following questions:   - Workshop 1: What are the different types of online eating disorder content (OEDC) that you think young people might engage with? What matters most to young people about OEDC? What terms/language might young people use when discussing this topic online? - Workshop 2: Looking at the draft list of themes that we have found, is there anything which you think is really important, or less important, is there anything you were surprised by, or is there anything missing?   An interactive white board was used to encourage the anonymous posting and analysis of ideas. Participants were also encouraged to use the meeting software’s chat function and their microphones.  Two of the young people from the workshops agreed to continue working as part of the research team after the workshops (as “young advisors”). They contributed to the synthesis towards its end. At this point, four new includable studies had been found and these studies needed to be incorporated in the synthesis. These two read these four papers and the draft write up of the synthesis of the studies previously included. They shared their ideas, along with the rest of the research team, about how the findings in the four new studies might complement or be distinct from the themes already present in the write up of the synthesis.  These two young advisors became authors of this paper, contributing to various points of the text in addition to the work described above. These written contributions included a fuller description of the value of a qualitative evidence synthesis for this review’s topic area, additional detail in the methods to describe their own involvement, descriptive detail to help characterise the set of included studies, as well as proposed responses to some of the peer reviewers’ comments on the paper’s first draft. |
| **Review stages** | |
| **Defining inclusion and exclusion criteria** | To be included in the qualitative evidence synthesis, studies needed to meet the following criteria:   - **Language and date**: studies needed to be published from 2007. - **Geographical location**: conducted in an OECD country. - **Population:** sample is of young people who identify as having had an eating disorder, disordered eating or eating difficulties, and their mean age is between 11 to 25 years old. - **Topic and study design:** qualitative studies exploring young people’s experience of online eating disorder content   The criteria are set out in full in **Supplementary File A:** **Table 2** below. This includes the criteria for the larger systematic map that preceded screening for inclusion in the QES. |
| **Search strategy** | Searches were undertaken in 15 health, psychology and education databases: ASSIA (Proquest); British Education Index (EBSCO); CINAHL PLUS (EBSCO); Child Development and Adolescent Studies (EBSCO); ERIC (EBSCO); EMBASE (OVID); IBSS (Proquest); MEDLINE (OVID); PsycINFO (OVID); Scopus, Social Policy and Practice (OVID); Sociological Abstracts (Proquest); and Web of Science databases: Book Citation Index- Social Science; Emerging Sources Citation Index; and Social Science Citation Index.  We also searched the following online resources: Mental Elf, NICE Evidence Search, Schools Health Education Unit website, UK Safer Internet Centre website, focused searches of: Bielefeld Academic Search Engine (BASE), Google, Google Scholar, Microsoft Academic. Focused searches were also undertaken within the journal pages of Appetite, Eating Disorder Journal, the Journal of Eating Disorders and the International Journal of Eating Disorders and citation searching, reference checking and related item searching of included qualitative studies and relevant systematic reviews, using Microsoft Academic. We also invited the stakeholder advisory group to provide any references they knew of.  The searches were undertaken between February and early March 2021 and a check for more recent studies was made using the same searches in October 2024. The searches were based on search concepts of: 1) disordered eating, body dissatisfaction or over-exercising symptoms and behaviours, 2) online content, social media, blogs, instant chat 3) adolescents and young adults. The search terms were developed by an information specialist in consultation with the lead author, and included pilot screening of a sample of 350 references identified from broad and focused scoping searches of which 59 were potentially relevant. We also consulted previous systematic reviews. The search strategy for MEDLINE is set out as **Supplementary File A:** **Box 1** below; this was translated for searches of other sources. |
| **Study selection** | Search results were imported into the systematic review software, EPPI-Reviewer and de-duplicated. We piloted the exclusion criteria by comparing decisions in groups of two or more reviewers using worksheets with guidance notes on a sample of 10 records. Disagreements were resolved through consensus and any required refinements to the criteria were made and recorded in a working protocol document. A further sample of records were screened by reviewers independently and differences resolved by discussion or consulting with a third reviewer. When agreement was adequate (90-95%) for this second sample, the remaining citations were screened by a single reviewer. References were screened initially on titles and abstracts and full reports were obtained for those references judged as meeting the inclusion criteria or where there was insufficient information from the title and abstract to assess relevance. A second opinion was made available for any study where a reviewer was unsure of its eligibility. |
| **Data extraction** | The coding tool used to extract descriptive data from studies was developed by building on an existing tool which captured generic information (e.g. date, geographical location, study design, population details, and factors studied) (Dickson et al. 2018). This tool was piloted with a random sample of 20 studies and additional dimensions of potential interest were added. Based on testing a further 10 studies the coding tool was refined and guidance was developed to help reviewers understand each dimension and apply the correct codes (see **Supplementary File A:** **Table 3** below). Data were extracted from full texts using EPPI-Reviewer. |
| **Quality appraisal** | Each study was critically appraised against eight questions (see **Supplementary File A: Box 2**). These were modified from a set of criteria developed for examining the findings of evaluations of intervention processes in a review of behavioural interventions for sexually transmitted diseases in young people (Shepherd et al., 2010). The criteria were based on previous work at the EPPI-Centre on assessing the quality of qualitative research and process evaluations (Harden 2007a, b), as well as the work of others in the field (Popay et al. 2003).  The Box shows the individual questions and responses available for each appraisal question, along with guidance for reviewers. As a final step in the study assessment, reviewers assigned to the studies ratings for two types of ‘weight of evidence’: reliability and usefulness. First, they assigned a weight (low, medium or high) to rate the reliability or trustworthiness of the findings (the extent to which the methods employed were rigorous thus avoiding bias and error). The reliability weighting focused on criteria 1 to 4. Secondly, they assigned a weight (low, medium, high) to rate the usefulness of the findings (how rich and complex the description and analysis of children’s views was in a study’s findings and whether or not the data threw light on children’s own explanations around body size). The usefulness weighting focused on the criteria numbered 5 and 6. The breadth and depth of the findings were considered in relation to their ability to answer our review question, as opposed to the aims and objectives of each individual study. No studies were excluded from the synthesis on the basis of their assessment weightings. |
| **Synthesis** | Data was synthesised thematically. Qualitative data (both direct participant quotes and primary study authors’ interpretations) in the studies’ findings sections were coded line-by-line in EPPI-Reviewer. The coding of that data was then organised into themes and higher-order themes. At the start of this process studies were organised by whether the participants had lived experience of eating disorders or not, to inform two separate but complementary syntheses. This paper reports on the synthesis of studies of young people with lived experience of eating disorders only. This synthesis was conducted first.  The thematic approach to synthesis is particularly suitable for systematic reviews, because the discipline of line-by-line coding requires that reviewers consider carefully each aspect of every study; and whether or not a finding from one study really does ‘translate’ into another (Thomas and Harden, 2008). It is considered less overtly dependent than several other synthesis methods on the theories and methodology that underpin included studies, which can vary considerably (Booth et al., 2018). This approach also enables findings and new conceptualisations to emerge inductively from the included studies, and so is a good fit with one of the principles of our review which aimed to highlight young people’s own perspectives. |
| **Information management** | |
| **Review management and reporting** | The review data were managed using the specialist reviewing software EPPI-Reviewer 4 (Thomas et al., 2010). A record of the screening process was captured using a PRISMA flow diagram (see **Supplementary File A:** **Figure 1** below). This report adheres to the Preferred Reporting Items for Systematic Reviews and Meta Analyses (PRISMA) Checklist (see **Supplementary File A:** **Figure 2** below). |

**References for works cited in Supplementary File A: Table 1**

Booth A, Noyes J, Flemming K, Gerhardus A, Wahlster P, van der Wilt GJ, Mozygemba K, Refolo P, Sacchini D, Tummers M, Rehfuess E (2018) Structured methodology review identified seven (RETREAT) criteria for selecting qualitative evidence synthesis approaches. Journal of clinical epidemiology; 99:41-52.

Dickson K, Richardson M, Kwan I, MacDowall W, Burchett H, Stansfield C, Brunton G, Sutcliffe K, Thomas J (2018) Screen-based activities and children and young people’s mental health: A Systematic Map of Reviews, London: EPPI-Centre, Social Science Research Unit, UCL Institute of Education, University College London.

Harden A (2007a) Does study quality matter in systematic reviews that include qualitative research? Paper presented at: XV Cochrane Colloquium, Sao Paulo, Brazil, 23-27 October.

Harden A (2007b) The quality of qualitative evidence: a review of assessment tools. Paper presented at: Seventh Annual International Campbell Colloquium, London, 14-16 May.

Popay J, Arai L, Roberts H, Roen K (2003) Preventing accidents in children: how can we improve our understanding of what really works? London: Health Development Agency

Shepherd J, Kavanagh J, Picot J, Cooper K, Harden A, Barnett-Page E, Jones J, Clegg A, Hartwell D, Frampton G, Price A (2010) The effectiveness and cost-effectiveness of behavioural interventions for the prevention of sexually transmitted infections in young people aged 13 to 19: a systematic review and economic evaluation. Health Technology Assessment; 14 (7): 1-206.

Thomas J, Harden A. Methods for the thematic synthesis of qualitative research in systematic reviews (2008) BMC medical research methodology ;8(1):1-10.

Thomas J, Brunton J, Graziosi S (2010) EPPI-Reviewer 4.0: software for research synthesis. EPPI-Centre software. London: Social Science Research Unit, Institute of Education, University of London.
