# Supplementary material for: The complexities and contradictions of online eating disorder content: A qualitative evidence synthesis of young people’s views and experiences
Source: PLOS Digit Health. 2026 Oct 9;5(10):e0001066. doi: 10.1371/journal.pdig.0001066 (PMC13652209; doi:10.1371/journal.pdig.0001066)
Supplement: Supplementary file 1 [file pdig.0001066.s001.zip › S1_Appendix/BoxB.docx]

**S1 Box B**

Criteria used to appraise study quality*

*Criterion* (with guidance for reviewers)

1. Were steps taken to increase rigour in the sampling?

Consider whether:

- The sampling strategy was appropriate to the questions posed in the study (eg, was the strategy well reasoned and justified?);
- Attempts were made to obtain a diverse sample of the population in question (think about who might have been excluded; who may have had a different perspective to offer);
- Characteristics of the sample critical to the understanding of the study context and findings were presented (ie, do we know who the participants were in terms of, eg, basic sociodemographics, characteristics relevant to the context of the study, etc).

2. Were steps taken to increase rigour in the data collected?

Consider whether:

- Data collection tools were piloted/(and if quantitative) validated;
- (If qualitative) data collection was comprehensive, flexible and/or sensitive enough to provide a complete and/or vivid and rich description of people’s perspectives and experiences (eg, did the researchers spend sufficient time at the site/with participants? Did they keep ‘following up’? Was more than one method of data collection used?);
- Steps were taken to ensure that all participants were able and willing to contribute (eg, processes for consent, language barriers, power relations between adults and children/young people).

3. Were steps taken to increase rigour in the analysis of the data?

Consider whether:

- Data analysis methods were systematic (eg, was a method described/can a method be discerned?);
- Diversity in perspective was explored;
- (If qualitative) the analysis was balanced in the extent to which it was guided by preconceptions or by the data);
- The analysis sought to rule out alternative explanations for findings (in qualitative research this could be carried out by, eg, searching for negative cases/exceptions, feeding back preliminary results to participants, asking a colleague to review the data, or reflexivity; in quantitative research this may be carried out by, eg, significance testing).

4. Were the findings of the study grounded in/ supported by the data?

Consider whether:

- Enough data are presented to show how the authors arrived at their findings;
- The data presented fit the interpretation/support claims about patterns in data;
- The data presented illuminate/illustrate the findings;
- (For qualitative studies) quotes are numbered or otherwise identified and the reader can see that they don’t just come from one or two people.

**Figure 1 continued**

5. Please rate the findings of the study in terms of their breadth and depth.

Consider whether:

- (NB: it may be helpful to consider ‘breadth’ as the extent of description and ‘depth’ as the extent to which data has been transformed/analysed);
- A range of issues are covered;
- The perspectives of participants are fully explored in terms of breadth (contrast of two or more perspectives) and depth (insight into a single perspective);
- Richness and complexity has been portrayed (eg, variation explained, meanings illuminated);
- There has been theoretical/conceptual development.

6. To what extent does the study privilege the perspectives and experiences of young people?

Consider:

- Whether there was a balance between open-ended and fixed response options;
- Whether children were involved in designing the research;
- Whether there was a balance between the use of an a priori coding framework and induction in the analysis;
- The position of the researchers (did they consider it important to listen to the perspectives of children?);
- Whether steps were taken to assure confidentiality and put young people at ease.

7. Overall, what weight would you assign to this study in terms of the reliability/trustworthiness of its findings? *Guidance: Think (mainly) about the answers you have given to questions 1 to 4 above.*

8. What weight would you assign to this study in terms of the usefulness of its findings for this review?

- Guidance: Think (mainly) about the answers you have given to questions 5 and 6 above and consider:
- The match between the study aims and findings and the aims and purpose of the synthesis;
- Its conceptual depth/explanatory power.

*****The following responses were available for these questions: (1–3) Yes, a fairly thorough attempt was made; Yes, several steps were taken; Yes, a few steps were taken; No, not at all/Not stated/Can’t tell. (4) Good grounding/support; fair grounding/support; limited grounding/support. (5) Limited breadth or depth; good/fair breadth but very little depth; good/fair depth but very little breadth; good/fair breadth and depth. 6. Not at all; a little; somewhat; a lot.
