# Supplementary material for: The complexities and contradictions of online eating disorder content: A qualitative evidence synthesis of young people’s views and experiences
Source: PLOS Digit Health. 2026 Oct 9;5(10):e0001066. doi: 10.1371/journal.pdig.0001066 (PMC13652209; doi:10.1371/journal.pdig.0001066)
Supplement: Supplementary file 1 [file pdig.0001066.s001.zip › S1_Appendix/PRISMA_Checklist.pdf]

S1: PRISMA Checklist

Section/topic	#	Checklist item	Reported in section #
**TITLE**			
**Title**	1	Identify the report as a systematic review, meta-analysis, or both.	Title
**ABSTRACT**			
**Structured summary**	2	Provide a structured summary including, as applicable: background; objectives; data sources; study eligibility criteria, participants, and interventions; study appraisal and synthesis methods; results; limitations; conclusions and implications of key findings; systematic review registration number.	Abstract<br>Some of the listed items are presented only in the full body of text. For example, limitations are explored only in discussion section
**BACKGROUND**			
**Rationale**	3	Describe the rationale for the review in the context of what is already known.	Introduction
**Objectives**	4	Provide an explicit statement of questions being addressed with reference to participants, interventions, comparisons, outcomes, and study design (PICOS).	Introduction
**METHODS**			
**Protocol and registration**	5	Indicate if a review protocol exists, if and where it can be accessed (for example, web address), and, if available, provide registration information including registration number.	CRD249969
**Eligibility criteria**	6	Specify study characteristics (for example, PICOS, length of follow-up) and report characteristics (for example, years considered, language, publication status) used as criteria for eligibility, giving rationale.	Methods and Supplementary file A
**Information sources**	7	Describe all information sources (for example, databases with dates of coverage, contact with study authors to identify additional studies) in the search and date last searched.	Methods and Supplementary file A
**Search**	8	Present full electronic search strategy for at least one database, including any limits used, such that it could be repeated.	Methods and Supplementary file A
**Study selection**	9	State the process for selecting studies (i.e., screening, eligibility, included in systematic review, and, if applicable, included in the meta-analysis).	Methods and Supplementary file A
**Data collection process**	10	Describe method of data extraction from reports (for example, piloted forms, independently, in duplicate) and any processes for obtaining and confirming data from investigators.	Methods and Supplementary file A
**Data items**	11	List and define all variables for which data were sought (for example, PICOS, funding sources) and any assumptions and simplifications made.	n/a

Section/topic	#	Checklist item	Reported in section #
**Risk of bias in individual studies**	12	Describe methods used for assessing risk of bias of individual studies (including specification of whether this was done at the study or outcome level), and how this information is to be used in any data synthesis.	Methods and Supplementary file A
**Summary measures**	13	State the principal summary measures (for example, risk ratio, difference in means).	n/a
**Synthesis of results**	14	Describe the methods of handling data and combining results of studies, if done, including measures of consistency (for example, I^2) for each meta-analysis.	Methods and Supplementary file A (no meta-analysis)
**Risk of bias across studies**	15	Specify any assessment of risk of bias that may affect the cumulative evidence (for example, publication bias, selective reporting within studies).	n/a
**Additional analyses**	16	Describe methods of additional analyses (for example, sensitivity or subgroup analyses, meta-regression), if done, indicating which were pre-specified.	n/a
**RESULTS**			
**Study selection**	17	Give numbers of studies screened, assessed for eligibility, and included in the review, with reasons for exclusions at each stage, ideally with a flow diagram.	Results and Supplementary file A
**Study characteristics**	18	For each study, present characteristics for which data were extracted (for example, study size, PICOS, follow-up period) and provide the citations.	Results, Table 1 and Supplementary file B
**Risk of bias within studies**	19	Present data on risk of bias of each study and, if available, any outcome level assessment (see item 12).	Results, Table 2
**Results of individual studies**	20	For all outcomes considered (benefits or harms), present, for each study: (a) simple summary data for each intervention group (b) effect estimates and confidence intervals, ideally with a forest plot.	Supplementary file B
**Synthesis of results**	21	Present results of each meta-analysis done, including confidence intervals and measures of consistency.	Results
**Risk of bias across studies**	22	Present results of any assessment of risk of bias across studies (see Item 15).	n/a
**Additional analysis**	23	Give results of additional analyses, if done (for example, sensitivity or subgroup analyses, meta-regression [see Item 16]).	n/a
**DISCUSSION**			
**Summary of evidence**	24	Summarise the main findings including the strength of evidence for each main outcome; consider their relevance to key groups (for example, healthcare providers, users, and policy makers).	Discussion
**Limitations**	25	Discuss limitations at study and outcome level (for example, risk of bias), and at review-level (for example, incomplete retrieval of identified research, reporting bias).	Discussion
**Conclusions**	26	Provide a general interpretation of the results in the context of other evidence, and implications for future research.	Discussion
**FUNDING**			

Section/topic	#	Checklist item	Reported in section #
**Funding**	27	Describe sources of funding for the systematic review and other support (for example, supply of data); role of funders for the systematic review.	Reported in submission

Where necessary, the PRISMA guidance has been adapted to fit with a systematic review of qualitative rather than quantitative date.

Where checklist items were not applicable to a systematic review of qualitative evidence, these were marked as not applicable (N/A).

Supplementary file X. Completed PRISMA checklist. The PRISMA checklist is reproduced from Moher et al. (2009) under the Creative Commons Attribution 4.0 International Licence (CC BY 4.0; https://creativecommons.org/licenses/by/4.0/). Items not applicable to this systematic review of qualitative evidence are marked N/A.
