# Supplementary material for: The complexities and contradictions of online eating disorder content: A qualitative evidence synthesis of young people’s views and experiences
Source: PLOS Digit Health. 2026 Oct 9;5(10):e0001066. doi: 10.1371/journal.pdig.0001066 (PMC13652209; doi:10.1371/journal.pdig.0001066)
Supplement: Supplementary file 1 [file pdig.0001066.s001.zip › S1_Appendix/TableB.docx]

**S1 Table B**

Inclusion criteria

|  | Include studies: | Exclude studies: |
| --- | --- | --- |
| A. Criteria for inclusion in the systematic map. | | |
| Participants | Young people between 11 and 25 years old. | Mean population age is outside the 11-25 age range. |
| Topic | Online eating disorder content | Not focused on online ED content |
| Outcomes [quantitative studies only] | Quantitative measures of: eating disorder symptoms, weight control behaviours or body image | Not reporting quantitative data on eating disorder symptoms, weight control behaviours or body image |
| Study Design (for map) | i) Observational studies including prospective cohort and case – control studies and randomised controlled trials (if available). | Not measuring the association between engagement with online eating disorder content and differences in eating disorder symptoms, weight control behaviours or body image; |
|  | ii) Qualitative studies of young people’s views and experiences | Not reporting qualitative data collection methods, analysis or empirical data (for example quotes/author description) on online eating disorder content. |
| Geographical Location | Conducted in an OECD Country | Not conducted in an OECD country |
| Date | 2007 and after | Pre-2007 |
| Language | Published in English | Not published in English |
| Availability | Electronically available in full text | Paper-based / hard copies of studies only |
| B. Additional criteria for inclusion in the Qualitative Evidence Synthesis | | |
| Study Design  (for QES) | Qualitative studies of young people’s views and experiences | Not reporting qualitative data collection methods, analysis or empirical data (for example quotes/author description) on online eating disorder content |
| Population  (for QES) | Young people identifying as having had an eating disorder, disordered eating or difficulties with eating. | Young people not identifying as having had an eating disorder, disordered eating or difficulties with eating. |
