# Supplementary material for: The complexities and contradictions of online eating disorder content: A qualitative evidence synthesis of young people’s views and experiences
Source: PLOS Digit Health. 2026 Oct 9;5(10):e0001066. doi: 10.1371/journal.pdig.0001066 (PMC13652209; doi:10.1371/journal.pdig.0001066)
Supplement: Supplementary file 1 [file pdig.0001066.s001.zip › S1_Appendix/BoxA.docx]

**S1 Box A**

Medline search strategy

Database: Ovid MEDLINE(R) and Epub Ahead of Print, In-Process, In-Data-Review & Other Non-Indexed Citations and Daily <1946 to February 19, 2021>

Search Strategy:

--------------------------------------------------------------------------------

1 blogging/ or social media/ or internet/ or online social networking/ (83311)

2 social networking/ and (online or web or internet or website* or computer? or electronic or computing or mobile or digital).ti,ab,kw,oa. (1466)

3 (instagram* or facebook* or Twitter or reddit or pinterest or YouTube* or MySpace or Tiktok or Tumblr or 4chan or imgur or "telegram messenger" or snapchat or whatsapp or imessage or wechat or viber or skype or flickr or linkedin or badoo or bebo or Cyworld or Orkut or Renren or Vkontakte or Friendster or "see you too" or "CU2" or "friends reunited" or "telegram messenger").ti,ab,kw,oa. (23291)

4 (blogs or blogger or blogging or bloggers or microblog* or "micro blog*" or "web log*" or weblog* or "web-based log" or vlog* or "videoblog*").ti,ab,kw,oa. (1806)

5 ("online chat" or chatroom* or chatbot* or "chat bot" or "chat bots" or "chat room" or "chat rooms" or "chat applications" or "chat app" or "chat apps" or "social media" or "digital media" or cyberspace or cyberculture or "web 2.0" or "social networking site" or "social networking sites" or "social web*" or retweet or tweet or tweeting).ti,ab,kw,oa. (17737)

6 ("computer mediated communication" or (((instant or mobile or online or "on line" or digital or apps or app or applications or software) adj2 (messaging or messenger)) or "social network site" or "social network sites" or ((instant or mobile or online or "on line" or digital or apps or app) adj2 communication) or "social computing")).ti,ab,kw,oa. (2971)

7 ((online or "on line" or web or internet or digital or website* or "new technolog*" or "new media" or virtual or cyber?) adj3 (forum or forums or network* or community or communities or social or socially or interact* or platform or platforms or chat or participat* or world or membership or communication)).ti,ab,kw,oa. (32182)

8 ((online or "on line" or web or internet or digital or website* or "new technolog*" or "new media" or virtual or cyber?) adj3 (users or engage or interact* or selfies or post or posts or posting or clicking or commenting or following or follower* or engaging or engagement or engag* or sharing or share or endorsing or endorse or tagging or tag or repost or reposts or reposting or tweet or retweet or contributing or contribute* or (content adj1 generat*) or (group adj1 membership*) or (passive* adj1 scrolling) or friending)).ti,ab,kw,oa. (14766)

9 ((online adj2 content) or (online adj2 harms) or (online adj2 exposure*) or ("on line" adj2 content) or ("on line" adj2 harms) or ("on line" adj2 exposure*) or ((online or "on line") adj3 (sites or sites))).ti,ab,kw,oa. (1109)

10 (((website* or web or internet) adj10 (harms or exposure*)) or ("web sites" or websites or "web page*" or "internet site" or "internet sites" or "internet pages") or ((website* or web or internet) adj10 (content not "content analysis"))).ti,ab,kw,oa. (20046)

11 1 or 2 or 3 or 4 or 5 or 6 or 7 or 8 or 9 or 10 (147012)

12 ("anti ED" or "anti eating*" or "antieating*" or "anti mia" or "anti ana" or "Anti Pro Anore*" or "pro eating" or Proeating or "e ana" or "pro ana" or "pro mia" or "pro ED" or "e mia" or "pro anorex*" or "pro anorec*" or proanorec* or "anti bulim*" or "pro bulim" or probulim* or antibulim* or "e bulim*").ti,ab,kw,oa. (212)

13 (thinspiration or fitspiration* or bonespiration* or ?thinspiration* or ?fitspiration* or ?bonespiration* or ?cheatmeal* or malefitspo* or ?malefitspo* or "thin inspiration*" or "body inspiration*" or "fitness inspiration" or "bone inspiration*" or "cheat meal*" or Bopo or fitspo or ?fitspo or ?bopo or fitstagram*).ti,ab,kw,oa. (87)

14 ("body positive" adj10 (content or online or "on line" or web or website* or internet or media)).ti,ab,kw,oa. (10)

15 ((Body* or eating or "food intake" or appetite or muscle) adj3 (Dysfunc* or Dysmorph* or disorder* or disfunction*) adj10 (content or online or "on line" or web or website* or internet or media)).ti,ab,kw,oa. (668)

16 ((anorexi* or bulimic or bulimia or boulimia or bulimarexia or diabulimia or bigorexia or orthorexia or pica or "Adonis complex" or manorexia or drunkorexia or "muscle dysmorphia" or EDNOS or EDNS or ARFID or "food neophobia" or "food addiction" or "addictive eating" or "night eating syndrome*" or "compulsive eating" or "body shame" or "athlete triad syndrome" or "rumination syndrome" or "rumination syndromes" or "rumination disorder*" or merycism) adj10 (content or online or "on line" or web or website* or internet or media)).ti,ab,kw,oa. (371)

17 12 or 13 or 14 or 15 or 16 (1177)

18 (anorexi* or bulimic or bulimia or boulimia or bulimarexia or diabulimia or bigorexia or orthorexia or pica or "Adonis complex" or manorexia or drunkorexia or "muscle dysmorphia" or EDNOS or EDNS or ARFID or "food neophobia" or "food addiction" or "addictive eating" or "night eating syndrome*" or "compulsive eating" or "body shame" or "athlete triad syndrome" or "rumination syndrome" or "rumination syndromes" or "rumination disorder*" or merycism or ((Body or eating or "food intake" or appetite or muscle) adj3 (Dysfunc* or Dysmorph* or disorder* or disfunction*))).ti,ab,kw,oa. (67926)

19 (ED adj1 (symptom* or diagnos* or development or behav*)).ti,ab,kw,oa. (1665)

20 Dysmorphophobia.ti,ab,kw,oa. (180)

21 ("body image" adj3 (problem* or disorder* or disturb* or distort* or disfunction* or dysfunction* or perception*)).ti,ab,kw,oa. (2680)

22 Body Dissatisfaction/ or Body Dysmorphic Disorders/ or Body Image/ or Body Image Disorders/ or Compulsive Behavior/ or "Feeding and Eating Disorders"/ or Bulimia Nervosa/ or

Somatoform Disorders/ or Anorexia Nervosa/ or "Avoidant Restrictive Food Intake Disorder"/ or "Binge-Eating Disorder"/ or Bulimia Nervosa/ or Diabulimia/ or "Food Addiction"/ or Night Eating Syndrome/ or Pica/ or "Relative Energy Deficiency in Sport"/ or Rumination Syndrome/ or Female Athlete Triad Syndrome/ or Body Composition/ or Thinness/ or Weight Lifting/px (105484)

23 18 or 19 or 20 or 21 or 22 (148397)

24 ("body positive" or "positive body" or "fitness movement" or "fitness culture" or "body talk" or "thin ideal" or "thinness ideal" or "body satisfaction" or "body attribute*").ti,ab,kw,oa. (2117)

25 (drive adj3 (thin or thinness or fitness or muscular*)).ti,ab,kw,oa. (1570)

26 (((body adj2 ideal*) or "body image" or "body weight" or muscular* or fitness) adj3 (content or exposure or images or imagery)).ti,ab,kw,oa. (1560)

27 ((negative or maladaptive) adj2 (body or eating or exercise or exercising or "weight control")).ti,ab,kw,oa. (3546)

28 ((excess* or problem* or atypical or harmful* or harms or disorder*) adj3 (exercise or exercising or dieting or diet or diets or eat or eating or purge or purging or vomit* or fasting or ruminating or fitness or "weight control")).ti,ab,kw,oa. (28241)

29 ((dissatisif* or anxiety or distress or concerns or fear) adj5 (body or appearance or fat or thin or thinness or muscular* or eating)).ti,ab,kw,oa. (7450)

30 (esteem adj5 (body or appearance or acceptance)).ti,ab,kw,oa. (1834)

31 (appreciation adj5 (body or appearance)).ti,ab,kw,oa. (333)

32 ((perception* or understanding or comprehension) adj5 (body or appearance or acceptance or fat or overweight or underweight or fitness or thin or thinness or muscular*)).ti,ab,kw,oa. (9430)

33 ((restrictive or disturbed or disordered or controlled or controlling or addiction or addictive or constrained or restrained or restraining or concerns or harms or compulsive or compulsion or compulsive or unconstrained or uncontrolled or binge or bingeing or addiction or addictive or intention or unrestrained or obsessive or preoccupied or preoccupied) adj3 (food or exercise or eating or exercising or diet or dieting or diets or fasting or purging or vomit* or "weight control")).ti,ab,kw,oa. (21369)

34 ((internalis* or "pre occupation" or "pre occupied" or "over concern" or rumination or ruminating) adj3 (body or muscular* or fat or thin or thinness or weight or eating or exercise or exercising)).ti,ab,kw,oa. (317)

35 ("over eat*" or "under eat*" or overeat* or undereat*).ti,ab,kw,oa. (3394)

36 ((unhealthy or choice* or behav* or beliefs or attitud* or motivation or motivations or patterns or psycholog* or psychopathology or symptom* or pathology*) adj3 (dieting or eating or purging or vomit* or ruminating or fasting or purge or fitness or "body fat" or exercise or exercising or "weight control")).ti,ab,kw,oa. (38600)

37 ("Exercise dependence" or "Food avoidance" or "dietary restraint").ti,ab,kw,oa. (1794)

38 24 or 25 or 26 or 27 or 28 or 29 or 30 or 31 or 32 or 33 or 34 or 35 or 36 or 37 (90213)

39 child/ or adolescent/ or young adult/ or students/ or Adolescent Behavior/ or Adolescent Health/ (3294540)

40 ("young people*" or schoolchild* or adolescen* or youth* or "young person*" or teen* or ((emerging or emergent or young or transition*) adj2 adult*) or (Young adj2 (men or "men's" or women* or female or females or male or males or user or users)) or (Millennial* or "college students" or undergraduate* or freshmen* or freshman* or Sophomore* or "generation Y")).ti,ab,kw,oa. (609291)

41 (children or boys or girls or students).ti,ab,kw,oa. (1355137)

42 adolescen*.jw. (47556)

43 39 or 40 or 41 or 42 (3974246)

44 23 or 38 (206458)

45 11 and 44 (1933)

46 43 and 45 (1037)

47 17 or 46 (1975)

48 limit 47 to yr="2007 -Current" (1711)

49 limit 48 to english language (1636)

50 limit 49 to (comment or letter or news) (8)

51 49 not 50 (1628)

52 animals/ not (animals/ and humans/) (4757437)

53 51 not 52 (1599)
