# Supplementary material for: The complexities and contradictions of online eating disorder content: A qualitative evidence synthesis of young people’s views and experiences
Source: PLOS Digit Health. 2026 Oct 9;5(10):e0001066. doi: 10.1371/journal.pdig.0001066 (PMC13652209; doi:10.1371/journal.pdig.0001066)
Supplement: Supplementary file 1 [file pdig.0001066.s001.zip › S1_Appendix/TableC.docx]

**S1 Table C**

Data extraction tool

| **Type of Online Content** | |
| --- | --- |
| Body Image Ideals | **Thin:** e.g. slim/slender bodies as socially desirable |
|  | **Fit:** e.g. toned/muscular bodies as socially desirable |
|  | **Beauty/attractiveness*:*** mainstream ideas about what makes a person beautiful and/or attractive |
| Body Positive | content which aims to challenge beauty ideals deemed socially desirable and encourage acceptance of a variety of body types |
| Eating disorder content | Any content online which encourages or discourages disordered eating e.g. pro-anorexia or pro-recovery |
| Health and Nutrition | Any content providing information on general health and/or nutrition |
| **Online platforms** | Studies were coded according to the type of platform where content was made available or originated from |
| **Levels of engagement** | |
| Observing | content and conversations from a distance but yet to engage further. E.g., largely 'viewing' content |
| Following | content, i.e. bookmarking websites or following or liking accounts (not posts) on social media. |
| Engaging | in content, e.g. clicking through to read, commenting on a posts, and/or searching for new content. |
| Endorsing | actively re-sharing content with others, i.e.  retweeting, re-posting via 'stories' or on personal walls. |
| Contributing | actively participating in content and interacting with others (i.e. posting in forums, Facebook groups. |
| **Population characteristics** | |
| Age | Studies were coded according to two mean age groups: 11-17 or 18-25 years old |
| Sex | Studies were coded according to whether they sampled boys/young men only, girls/young women only, or included mixed sex samples. |
| Clinical eating disorder diagnosis | Studies were coded according to whether study participants had a diagnosed eating disorder (yes, no or mixed sample) |
| Geographical location: | Studies were coded according to their geographical origin |
