# Supplementary material for: The complexities and contradictions of online eating disorder content: A qualitative evidence synthesis of young people’s views and experiences
Source: PLOS Digit Health. 2026 Oct 9;5(10):e0001066. doi: 10.1371/journal.pdig.0001066 (PMC13652209; doi:10.1371/journal.pdig.0001066)
Supplement: Supplementary file 2 [file pdig.0001066.s002.zip › S2_Appendix/TableA.docx]

**S2 Table A**

Reports of included studies identified and used in synthesis

|  | Main study report | Linked reports (other reports relating to the same study) |
| --- | --- | --- |
| 1 | Dashow, 2021 | - |
| 2 | Eikey & Booth, 2017 | Eikey & Reddy, 2017 |
| 3 | Faccio, 2024 | - |
| 4 | Firkins, 2017 | Firkins et al., 2019 |
| 5 | Hockin-Boyers et al., 2020 | Hockin-Boyers et al., 2021 |
| 6 | IPSOS, 2024 | - |
| 7 | Mincey, 2019 | Mincey & Hunnicut Hollerbaugh, 2022 |
| 8 | Nikolova & LaMarre, 2023 | - |
| 9 | Praet, 2024 | - |
| 10 | Saunders et al., 2020 | Saunders et al., 2018 |
| 11 | Sjöström, 2024 | - |
| 12 | Smahelova et al., 2020 | Drtilová et al., 2021 |
| 13 | Yeshua-Katz & Martin, 2013 | - |
