# Supplementary material for: The complexities and contradictions of online eating disorder content: A qualitative evidence synthesis of young people’s views and experiences
Source: PLOS Digit Health. 2026 Oct 9;5(10):e0001066. doi: 10.1371/journal.pdig.0001066 (PMC13652209; doi:10.1371/journal.pdig.0001066)
Supplement: Supplementary file 2 [file pdig.0001066.s002.zip › S2_Appendix/TableB.docx]

**S2 Table B**

Further detail from included studies

| **Author(s) (Year) Title  Study Focus** | **Aims & analysis types** | **Participant Characteristics** (where ages extended beyond 24 and age was made explicit in analysis, only data relating to participants aged up to 24 were used) | **Themes presented by study authors** |
| --- | --- | --- | --- |
| **Dashow, 2022**  “Food for thought: An exploration of the interplay between eating disorder recovery and recovery Instagram accounts”  **Study focus:**  Priori focus on ED type: **Yes** but not specific  Priori focus on ED related content: **Yes** recovery dedicated IG account  Priori focus on Digital platforms: **Yes** study focuses on active Instagram accounts dedicated to documenting recovery | The aim of the current study was to explore the interplay between individuals’ ED recovery and their recovery-based IG accounts. Twelve semi-structured interviews were conducted with individuals who have recovery-based IG accounts.  Analysis: Grounded Theory method. | **Country:** USA  **No of participants:** 12  **Age:** 18 – 31  **Gender:** female  **Ethnicity:** White (10) Hispanic (1) South Asian (1)  **SES:** Not stated  **Lived Experience of diagnosed with ED:**  History of ED, had recovery -dedicated IG account active for 6 months or more  **Other relevant details:**  11 participants heterosexual 1 identified as bisexual. Participants reported mixture of ED diagnoses: (6) anorexia (1) bulimia (1) other specified feeding/ED (4) history of both anorexia & bulimia. | - Not feeling sick enough - “I’ve been triggered by other people in the community” - “People are mindful of not triggering others” |
| **Eikey & Booth, 2017**  "Recovery and maintenance: How women with eating disorders use Instagram"  **Study focus:**  Priori focus on ED type: **Yes specific –** anorexia nervosa and bulimia nervosa and disordered eating behaviours related to these disorders because behaviours associated with these two types of eating disorders are similar  Priori focus on ED related content: **No**  Priori focus on Digital platforms: **Yes specific -**this study sheds light on how Instagram can exacerbate and improve eating disorder symptoms | The authors’ study differs in that they examine how women with eating disorders who interact with social media platforms, specifically, Instagram can use these interactions to aid recovery as well as exacerbate symptoms.  Analysis: Thematic Analysis (Braun & Clarke) | **Country:** USA  **No of participants:** 16  **Age:** 18-25  **Gender:** female  **Ethnicity:** White (12) Asian American or pacific islander (2) multi-racial (1) Native America (1)  **SES:** Not stated  **ED experience/diagnosis:** most reported being in recovery. ED from 2 months – 7 years. 4 out of 16 were diagnosed  **Other relevant details:** not stated | - Learn about the recovery process - Track recovery process - Learn about healthy food and exercises - Reduce stigma, increase awareness, and create a community for social support |
| **Faccio et al., 2024**  “Issues Related to the Use of Visual Social Networks and Perceived Usefulness of Social Media Literacy During the Recovery Phase: Qualitative Research Among Girls With Eating Disorders”  **Study focus:**  Priori focus on ED type: **Yes but not specific focus** is on end of eating disorder treatment  Priori focus on ED related content: **Yes** digital platforms are referred to looking at health risks and potential benefits.  Priori focus on Digital platforms: **Yes specific –**Instagram and Snap Chat | This study aims to examine how young women recovering from eating disorders in Northern Italy perceive the health risks and potential benefits associated with visual social networks (ie, Instagram and Snapchat). The literature has found these platforms to be detrimental to online body comparisons. It also explores the perceived usefulness, willingness, and personal interest in coconstructing social media literacy programs with girls recovering from eating disorders.  Analysis: Content analysis | **Country:** Italy  **No of participants: 30**  **Age:** 14-17  **Gender:** female  **Ethnicity**: not specified  **SES:** not specified  **ED experience/diagnosis:** a diagnosis of an ED, being in the early stages of treatment with at least 15 days of hospitalization, having an active Instagram and Snapchat profile for at least six months before admission, and willingness to participate in interviews.  **Other relevant details:** participants recruited though health care staff, authors were informed users of the local health unit for the treatment of EDs within the National Health Service in Eastern Veneto (specifically, the Casa delle Farfalle Residential Protected Therapeutic Community for Children). | - Social Network’s (SN) Active or Passive Ways of Use - Online Interactions’ Impact - Investment in the Photographic Dimension - Self-Representation - SNs’ Risks - Self-Protective Strategies While Using SNSs - SNs’ Potentials |
| **Firkins et al., 2017**  "Disordered Eating in a Digital Age: Narratives of Withdrawal from pro-ana/Mia Forums by Regular Site Users"  **Study focus:**  Priori focus on ED type: **Yes specific-** Anorexia stated as disorder of main interest in study's s aims (although bulimia is also referred to in the title, and pro-mia sites are referred to in participant info sheets)  Priori focus on ED related content: **Yes specific –** Both Pro-ana and Pro-mia sites are referred to in the interview participant info sheets, but the focus appears mainly to be on the former  Priori focus on Digital platforms: **Yes specific -** Main focus, from methods section, seems to be on forums. The background sections expand on this, mentioning: Blogs, LiveJournals or Vlogs on YouTube. Myspace, Facebook, Twitter, Flickr, Instagram, Pinterest and Tumblr | To address gaps in the pro-ana literature by conducting qualitative interviews with those who have regularly used pro-ana forums; with a focus on better understanding not only what led individuals to join this community but also how they storied the disengagement process and any impact it had on their lives"  From abstract: To "investigate[d] accounts of Pro-Ana withdrawal and its associated impact"  Analysis: Narrative (Work of Riessman) | **Country:** USA & UK  **No of participants:** 3 aged 18-24 (6 in study as a whole)  **Age:** 18+  **Gender:** female  **Ethnicity**: White British (3) African-America/black (4) Hispanic (3) Asian (3)  **SES:** educational level, at time of the study participants all in higher education All described (themselves p45) as middle class.  **ED experience/diagnosis:** onset reported from childhood (11-15) Official diagnosis was yes type not specified by doctor, but participant described as Anorexia, EDNOS  **Other relevant details:** participants needed to have regularly visited pro-ana sites in the past and have either reduced their use of them or stopped altogether. Pro-ana sites were first visited at age 14-15, all three participants aged 24 and under reported having been visiting daily at multiple times at the peak of their use, for between three and 25 hours/week. Out of the three, one had not posted/chatted to others on the site, two had (Table 1a). | - Driving Down a New Road: Coming to Ana - Driving Away from Pro-Ana: Withdrawal |
| **Hockin-Boyers et al., 2021** "#gainingweightiscool: the use of transformation photos on Instagram among female weightlifters in recovery from eating disorders".  **Study focus:**  Priori focus on ED type: **yes, but not specific transformation** photos of women in recovery  Priori focus on ED related content: **Yes, not specific**  we explore transformation photos on Instagram as 'digital artefacts'  Priori focus on Digital platforms: **Yes specific –** Instagram | By triangulating interview, photo elicitation and netnography data from research on female weightlifting as a tool for recovery from eating disorders, authors explore transformation photos in relation to three intersecting themes: 1) new modes of ‘becoming’, 2) representation and ‘mediated memories’, and finally, 3) survivorship and identity  Analysis: Interpretive Phenomenological Analysis (IPA) | **Country:** UK  **No of participants:** 19  **Age:** 17-36  **Gender:** female  **Ethnicity:** Not stated  **SES:** Not stated  **ED experience/diagnosis:** Female participants, aged 17 and over, living in the UK, who have a history of eating disorders and are weightlifting during their recovery.  **Other relevant details:** Not stated | - Social media, wellbeing and recovery from eating disorders - Digital pruning: the strategic design of social media ‘worlds’ |
| **IPSOS 2024** “Online Content: Qualitative Research. Experiences of children encountering online content relating to eating disorder, self harm and suicide**.”**  **Study focus:**  Priori focus on ED type: **Yes but not specific focus** self-defining with at least six months since discharge from an ED support service and without an active ED diagnosis  Priori focus on ED related content: **No not specified**  Priori focus on Digital platforms: **No not specified** | Ipsos UK and TONIC were commissioned to conduct qualitative research to understand the pathways through which children encounter online content that promotes suicide, self-harm and eating disorders. This research sought to build on the current evidence base by further exploring online content that professionals, children and young people perceive to promote suicide, self-harm and eating disorders, perceptions of how harmful this content can be and the impact it has on children. Pathways to encountering this content and perceptions of mitigations to prevent children from encountering this content were also explored.  Analysis not specified | **Country:** UK  **No of participants:** 8  **Age:** 3-21  **Gender:** female male  **Ethnicity:** Not stated  **SES:** Not stated  **ED experience/diagnosis:** sample broke down into self-reported lived experience of eating disorder and lived experience of eating disorder with self-harm  **Other relevant details:** Not stated | - Suicide, self-harm and eating disorder content - Pathways to encountering harmful content - Impact of encountering content promoting suicide, self-harm and eating disorders - Mitigations Safety Measures |
| **Mincey, 2019**  "An exploratory qualitative inquiry on the lived experiences of people who engage with pro-eating disorder online media  **Study focus:**  Priori focus on ED type: **yes, not specific**  Priori focus on ED related content: **Yes, specific**  Pro eating disorder  Priori focus on Digital platforms: **Yes, specific** Focus on online media webpages and applications/forums. Broader range mentioned in background “Webpages such as Tumblr, Instagram, Private or public online forums, …Facebook, Reddit…” | To investigate the meaning pro-eating disorder online media may have on individuals who use this form of social media. Ultimately, the objective of this study was to discover the voiced perceptions of those who engaged in pro-eating disorder online media, and their voiced purpose(s) of engaging in pro-eating disorder online media.  Analysis: Van Manen’s (1990, 2014) hermeneutic phenomenological approach | **Country:** USA  **No of participants:** 5 aged 24 or under (10 in study as a whole)  **Age:** 18-31  **Gender:** of the five aged under 24, 4 were female and 1 male  **Ethnicity:** Caucasian (3) African American (1) Biracial (1)  **SES:** some high school (3), college (2)  **ED experience/diagnosis:** Participants are not categorised using diagnosis. To be included in the study, participants needed to be accessing and using pro-eating disorder online media at the time of the study. All recounted experience with disordered eating or an ED.  **Other relevant details:** height and weight (current, highest, lowest and ideal), BMI and Scores of EAT – 26 and BAS-2, Scores of BDS. Location at time of study: US (n=6 – 4 of which aged 24 or under), Australia (n=1, aged 10), Canada, Ireland and Germany each with n=1 (all aged >24). Inclusion criteria were “…participants must be 18 years of age or older, and participants must be accessing and using pro-eating disorder online media at the time of the study ... Participants scores on the BDS ranged from 1 to 6 (M = 3.2, SD = 1.75). Four participants had scores of four or higher. According to Mutale et al. (2016), higher scores indicate a higher level of body dissatisfaction. Participants scores on the EAT-26 ranged from 7 to 50 with only two participants had an EAT-26 lower than 20. A score higher than 20 indicates a high level of concern about body weight and eating behaviours (Garner et al., 1982). Six participants had a 58 score over 40 on the EAT – 26 with three participants scoring a 50 (M = 36.6, SD | - curiosity - spiralling down - increased knowledge - unfulfilled needs |
| **Nikolova & LaMarre, 2023**  “If I Unfollow Them, Its Not a Dig at Them: A Narrative Analysis of Instagram Use in Eating Disorder Recovery”  **Study focus:**  Priori focus on ED type: **Yes but** nothing specific (Disordered eating)  Priori focus on ED related content: **Not specific** more broader health and fitness space  Priori focus on Digital platforms: **Yes** Instagram | The authors aimed to dig into the dynamics and tensions involved in experiences of Instagram use in ED recovery. Broadly, the research question was: What stories do women in New Zealand recovering from EDs tell about their social media experiences, particularly in using Instagram? Within this broad question, they sought to understand how Instagram use interacts with women’s experiences of ED recovery, and what downfalls and/or benefits women see in relation to Instagram use and ED recovery.  Analysis: Narrative thematic analysis – grounded in social constructionist paradigm | **Country:** New Zealand  **No of participants:** 7 aged 24 or under (8 in whole study)  **Age:** (7) 18 – 25 (1) late 40s  **Gender:** female  **Ethnicity:** all identified as Pakeha (New Zealand European)  **SES:** not stated  **Lived Experience of diagnosed with ED:**  Self-identify as in recovery from EDs (diagnosis not required)  **Other relevant details:** users of Instagram on a regular basis (3x per week). Being on a journey to recovery or being in recovery | - The Pitfalls of ED Recovery Accounts - Balancing On and Offline Engagement With Family and Friends - The Agency of Algorithms - The Limits of Instagram Body Positivity |
| **Praet et al., 2024**  “The Association of Social Media Use and Eating Behaviour of Belgian Adolescent Girls Diagnosed with Anorexia Nervosa—A Qualitative Approach”  **Study focus:**  Priori focus on ED type: **Yes specific focus on** diagnosed with anorexia nervosa (DSM V, restrictive type)**.**  Priori focus on ED related content: **No not specified**  Priori focus on Digital platforms: **No not specified** | This study aims to explore through qualitative semi-structured interviews whether adolescents with eating disorders perceive social media as influencing their illness.  Analysis: not specified by reference to coding framework and Axial coding | **Country:** Belgium  **No of participants:** 7  **Age:** 15-17  **Gender:** female  **Ethnicity:** Not stated  **SES:** Not stated  **ED experience/diagnosis:** female adolescents aged 12 to 17 diagnosed with anorexia nervosa (DSM V, restrictive type).  **Other relevant details:** Participants were recruited from the UZ Leuven paediatrics department by staff members. The paediatricians assessed the candidates’ eligibility for participation. Individuals for whom participation was deemed excessively challenging (due to clinical or psychological concerns) were excluded. | - selective content - biased interpretation - behavioural adaptation - recovery process |
| **Saunders et al., 2020**  "From self(ie)-objectification to self-empowerment: The meaning of selfies on social media in eating disorder recovery".  **Study focus:**  Priori focus on ED type: **yes, but not specific** "women self-identifying as in ED recovery"  Priori focus on ED related content: **yes, specific** Selfies – Analysis was of discussion of" selfies previously shared on social media and accompanying narrative"  Priori focus on Digital platforms: **Yes, specific** Instagram | Guided by objectification theory, to qualitatively examine the subjective role of selfies in young women’s ED recovery process [and]… to identify differences between subjectively helpful and subjectively harmful selfies in composition and content. The primary goal of the overarching study [involving 30 participants] was to examine the types, targets, and effects of social comparison for this population (Saunders and Eaton, 2018).  Analysis: Thematic analysis and (for photos) content analysis | **Country:** USA  **No of participants:** (27)  **Age:** 18-22  **Gender:** female  **Ethnicity:** African-America/black (4) Hispanic (3) Asian (3)  **SES:** upper-middle class/family income between $76,900 -$105,999  **ED experience/diagnosis:** not stated  **Other relevant details:** participants had Instagram accounts for an average of 6 yrs, spent 1-2 hours daily/checked every half hour – few hours. | - (Comparison with) ED self - (Comparison with) Peers, family, media - (Comparison with) Others in ED recovery or who currently appear unhealthy - (Comparison with) Societal & Medical Ideals |
| **Sjöström, 2024 et al., 2024**  Helpful or Not? A Qualitative Study on Female Adolescents’ Experience of TikTok When Recovering From Anorexia Nervosa  **Study focus:**  Priori focus on ED type: **Yes, specific type** anorexia nervosa  Priori focus on ED related content: **Yes specified** TikToks influence of adolescent's recovery from anorexia nervosa  Priori focus on Digital platforms: **Yes specified** Tik Tok | Drawing from the authors clinical experience that adolescents diagnosed with AN increasingly use TikTok in their recovery process, they aim to investigate how this app affects the outpatient recovery phase in AN. | **Country:** Sweden  **No of participants:** 14  **Age:** 13-17  **Gender:** female and male  **Ethnicity:** Not stated  **SES:** Not stated  **ED experience/diagnosis:** with a DSM- 5 diagnosis of AN, a minimum of 3 months of TikTok usage, and fluency in Swedish, excluding those with cognitive impairments  **Other relevant details:** Participants for this study were recruited from child and adolescent psychiatry (CAP) outpatient clinics for EDs in Malmö and Lund, Region Skåne, Sweden, in a convenience sampling fashion, through their health- care providers and information placed in waiting rooms. | - Social Connectivity: Immediate Support and Recovery Content - Algorithmic Engagement: Influence and Control - Regulation and Adaptation: Creating Safer Spaces - Personal Agency and Recovery Pathways |
| **Smahelova et al., 2020**  Internet Usage by Women with Eating Disorders during Illness and Recovery.  **Study focus:**  Priori focus on ED type: **Yes, specific type** frames interest as being in spectrum Anorexia, Bulimia, binge eating.  Priori focus on ED related content: **Not specific –** all types of internet.  Priori focus on Digital platforms: **Not specific all types were of interest websites** SNS blogs applications (including apps for smart phones) and online communities. | Authors’ aim are to present a complex view from the perspective of people with ED, and we propose the following research questions: Q1: Which online platforms and activities do women with eating disorders report? Q2: How do the identified platforms and activities fit into the three levels of the interactions model proposed by Rodgers (2016)? Q3: Which motivations to use specific online platforms do women with eating disorders report?  Analysis: Thematic Analysis (Braun & Clarke) | **Country:** Czech Republic  **No of participants:** (30)  **Age:** 16-28  **Gender:** female  **Ethnicity:** Not stated  **SES:** Not stated  **ED experience/diagnosis:** Participants needed to have experience (now or in the past) of a form of an eating disorder. Participants’ stated experiences were with: Anorexia Nervosa – AN N = 13, Bulimia Nervosa – BN N = 3, Binge Eating Disorder BED N = 1, or multiple ED diagnoses EDM N = 13. … either currently suffered with the ED and were in the recovery process (N = 22) or they had recovered (N = 8). [discussion notes all] had already received some kind of formal treatment for ED  **Other relevant details:** all were Czech | - Nonprofessional pro-ED platforms - Nonprofessional pro-recovery platforms - Platforms that allow counting calories and setting weight goals - Platforms about food, exercise, and lifestyle - Platforms providing professional help |
| **Yeshua-Katz & Martins, 2013**  "Communicating stigma: the pro-ana paradox"  **Study focus:**  Priori focus on ED type: **yes, specific** pro-ana if it included instruction for initiating and maintaining anorexia nervosa  Priori focus on ED related content: **Yes specific**  Authors interested in studying people who identified themselves as pro-anas. In order to meet this goal, sought out blogs that were clearly pro-ana sites.  Priori focus on Digital platforms: **Yes, specific** authors searched for pro-ana blogs via blog-hosting platforms such as blogger.com, Tumblr.com, and Livejournal.com. Pro-ana blogs were also found through blogrolls – that is, a list of links to other blogs that the author of one blog likes to read. To be included in the sampling frame, the blogs had to be updated at least once in the past six months (for example, Hu, 2009). Blogs presided over by mental health professionals, caregivers, or advocates were excluded. | The purpose of the study is to explore the motivations, benefits, and drawbacks of blogging about a stigmatised mental illness, as taken from the bloggers’ own perspective.  Analysis: Grounded Theory Technique (Charmaz 2006) | **No of participants:** (33)  **Age:** 15-33  **Gender:** female  **Ethnicity:** Caucasian (22) Mediterranean (2) African American (1) other (8)  **SES** Most of the participants were in high school (n=16) or attending college (n = 17)**.**  **ED experience/diagnosis:** The length of time most of the participants reported living with an ED was 6.8 years in spite of our efforts to include only bloggers who identified themselves as pro-ana (for example, defining anorexia as a lifestyle) as described in the published literature, participants expressed different approaches to their health condition. Indeed, most of the bloggers (n = 27) reported living with the disorder but six were in recovery. Twenty four bloggers defined their eating disorder as a mental illness, six bloggers defined their illness as a coping mechanism, and only three bloggers defined it as a lifestyle.  **Other relevant details:** Not stated | - Motives for Blogging - Benefits of Blogging - Drawbacks of Blogging |
